# Anomalous Magnetorheological Response for Carrageenan Magnetic Hydrogels Prepared by Natural Cooling

**DOI:** 10.3390/gels9090691

**Published:** 2023-08-28

**Authors:** Masahiro Kaneko, Mika Kawai, Tetsu Mitsumata

**Affiliations:** Graduate School of Science and Technology, Niigata University, Niigata 950-2181, Japan; f22b162a@mail.cc.niigata-u.ac.jp (M.K.); mikagoro@eng.niigata-u.ac.jp (M.K.)

**Keywords:** magnetic gel, stimuli-responsive gel, carrageenan, soft material, magnetorheological effect

## Abstract

The effect of the cooling rate on magnetorheological response was investigated for magnetic hydrogels consisting of carrageenan and carbonyl iron particles with a concentration of 50 wt.%. For magnetic gels prepared via natural cooling, the storage moduli at 0 and 50 mT were 3.7 × 10^4^ Pa and 5.6 × 10^4^ Pa, respectively, and the change in the modulus was 1.9 × 10^4^ Pa. For magnetic gels prepared via rapid cooling, the storage moduli at 0 and 50 mT were 1.2 × 10^4^ Pa and 1.8 × 10^4^ Pa, respectively, and the change in the modulus was 6.2 × 10^3^ Pa, which was 1/3 of that for the magnetic gel prepared by natural cooling. The critical strains, where *G*′ is equal to *G*″ on the strain dependence of the storage modulus, for magnetic gels prepared by natural cooling and rapid cooling, were 0.023 and 0.034, respectively, indicating that the magnetic gel prepared by rapid cooling has a hard structure compared to that prepared by natural cooling. Opposite to this, the change in the storage modulus at 500 mT for the magnetic gel prepared by rapid cooling was 1.6 × 10^5^ Pa, which was 2.5 times higher than that prepared by natural cooling. SEM images revealed that many small aggregations of the carrageenan network were found in the magnetic gel prepared by natural cooling, and continuous phases of carrageenan network with large sizes were found in the magnetic gel prepared by rapid cooling. It was revealed that magnetic particles in the magnetic gel prepared by rapid cooling can move and form a chain structure at high magnetic fields by breaking the restriction from the continuous phases of carrageenan.

## 1. Introduction

Stimuli-responsive soft materials [1,2,3,4,5] change their physical properties in response to stimuli such as light, temperature, and pH. Magnetic gels are among the stimuli-responsive soft materials, and their physical properties change in response to magnetic fields. Magnetic soft materials have attracted great attention as next-generation actuators because their physical properties change instantaneously and dramatically when a magnetic field is applied [6,7,8,9,10,11]. Magnetic hydrogels made of polysaccharides have been widely investigated thus far, and many functions and applications have been reported, such as recoverable adsorbent [12], drug delivery [13], and hyperthermia treatment [14]. These applications take advantage of the bio- and eco-friendly properties that are derived from natural products. In other words, magnetic gels made of natural products are harmless when they are taken into human body and do not cause pollution when diffused into the environment. When a magnetic field is applied to magnetic gels, the elastic modulus is higher compared to that without the magnetic field. This is called the magnetorheological effect (MR effect). The underlying mechanism is that magnetic particles come into contact with each other and form a chain-like structure.

Because magnetic particles strongly interact with the polymer network, they are subjected to compressive and tensile resistance forces from the polymer network when they are displaced in the gel. In other words, as this resistance force increases, it becomes more difficult for the magnetic particles to move within the gel, and a chain structure of magnetic particles is not formed. As a result, the amplitude of the MR effect is reduced. Actually, the MR effect decreases as the elastic modulus of the matrix increases in magnetic gels [11,15]. Magnetic gels with a matrix of natural polymers, such as carrageenan and agar gels, show a significant change in elastic modulus with the magnetic field compared to those with a matrix of synthetic polymers [16]. This suggests that the network structure of natural polymers allows magnetic particles to move more easily than that of synthetic polymers. This feature is considered to be due to the cross-linking points of natural polymers formed by hydrogen (physical) bonds. Since the force that magnetic particles receive from the magnetic field is much stronger than the binding force, the cross-linking points are broken when the magnetic particles move.

In our previous investigations, measured at a low magnetic field and low concentration of magnetic particles, it was shown that the change in storage modulus for carrageenan magnetic hydrogels took a maximum at around 2.0 wt.% [17]. This is anomalous behavior because it is normal that the change in modulus increases with decreasing polymer concentration. The decrease in the change in storage modulus is caused by increasing the strength of the carrageenan network, which was proved by increasing the critical strain. Conversely, we can find the strength of the carrageenan network from the critical strain.

In addition to this, there is some research describing how the physical properties for polysaccharide gels are strongly affected by the gelation speed, i.e., cooling rate. A stiff and heterogeneous gel structure with a high storage modulus is formed at a low cooling rate, and meanwhile a weak and homogeneous gel structure with a low storage modulus is formed at a high cooling rate [18]. It was also reported that the size of the junction zone (cross-linking point) decreased when a carrageenan solution cooled rapidly [19,20,21]. The changes in the structure of cross-linking points or the strength of cross-links might affect the magnetic response for magnetic gels. In this study, we investigated the effect of the cooling rate on the viscoelastic properties of carrageenan magnetic gels and their magnetic responses to clear the influence of cross-linking structures on the mobility of magnetic particles within the gel. It was revealed that the storage moduli for magnetic gels prepared at different cooling rates exhibited completely different magnetic field responses. It can be considered that this result reflects the difference in the network structure depending on the cooling rate, as reported in the literature [18].

## 2. Results and Discussion

**Figure 1:**
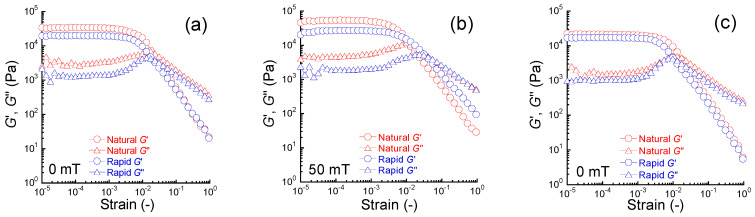
Strain dependence of storage and loss moduli for magnetic gels prepared by natural cooling and rapid cooling at (**a**) 0 mT and (**b**) 50 mT and for (**c**) carrageenan gels without magnetic particles at 0 mT (*f* = 1 Hz).

Figure 1a,b show the strain *γ* dependence of the storage modulus *G*′ and loss modulus *G*″ at 0 and 50 mT for magnetic gels prepared by natural cooling and rapid cooling, respectively. At 0 mT, regions of linear viscoelasticity were seen at strains below 1.7 × 10^−3^ and 2.4 × 10^−3^ in magnetic gels prepared by natural cooling and rapid cooling, respectively. The strains at which the *G*′ intersected with *G*″ were at 0.023 and 0.034 for magnetic gels prepared by natural cooling and rapid cooling, respectively. At *γ* = 1, the storage moduli for these magnetic gels showed the same value. At 50 mT, the *G*′ showed the linear viscoelastic region at strains below 1.3 × 10^−3^ and 2.4 × 10^−3^ for magnetic gels prepared by natural cooling and rapid cooling, respectively. The *G*′ crossed *G*″ at a strain of 0.016 for the naturally cooled magnetic gel and 0.052 for the rapidly cooled magnetic gel. An interesting behavior was observed at *γ* = 1, where the storage modulus for the rapidly cooled magnetic gel was higher than the naturally cooled one, which was the opposite of the result at low strains. This might be due to a structural change in large strains enabling the movement of magnetic particles. Figure 1c shows the strain dependence of *G*′ and *G*″ at 0 mT for carrageenan gels without magnetic particles (abbreviated as “carrageenan gel” hereafter) prepared by natural cooling and rapid cooling. Linear viscoelastic regions were observed at strains below 2.4 × 10^−3^ and 1.7 × 10^−3^ for carrageenan gels prepared by natural cooling and rapid cooling, respectively. The strains of *G*′ which crossed *G*″ were 0.014 and 0.013 for carrageenan gels prepared by natural cooling and rapid cooling, respectively. The parameters *G*′(*γ* = 1)/*G*′(*γ* = 10^−4^), representing the amplitude of the Payne effect [22], were 2.9 × 10^−4^ and 3.2 × 10^−4^ for carrageenan gels prepared by natural cooling and rapid cooling, respectively, and were almost the same.

**Figure 2:**
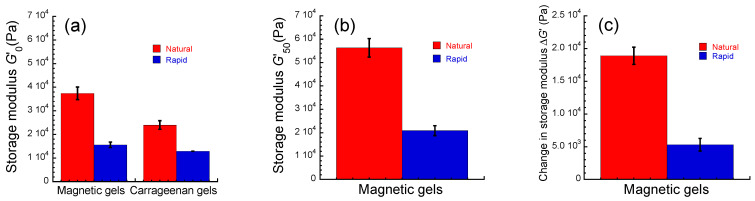
Storage moduli at (**a**) 0 mT and (**b**) 50 mT and (**c**) the change in the storage modulus for magnetic gels prepared by natural cooling and rapid cooling. Storage modulus for carrageenan gels without magnetic particles at 0 mT is also presented in Figure 2a (*f* = 1 Hz, *γ* = 10^−4^).

Figure 2a exhibits the storage modulus at 0 mT in the linear viscoelastic regime (*γ* = 10^−4^) for magnetic gels and carrageenan gels prepared by natural cooling and rapid cooling. The storage modulus for the magnetic gel prepared by natural cooling was 3.7 ± 0.3 × 10^4^ Pa, which is 2.3 times higher than that prepared by rapid cooling (=1.6 ± 0.1 × 10^4^ Pa). The storage modulus for the carrageenan gel prepared by natural cooling was 2.4 ± 0.2 × 10^4^ Pa, which is 1.8 times higher than that prepared by rapid cooling (=1.3 ± 0.0 × 10^4^ Pa). Accordingly, this indicates that a secondary structure of the carrageenan network with low elasticity was formed by rapid cooling. A similar phenomenon has been observed for gellan gum [18]. The storage modulus for the naturally cooled magnetic gel was 1.5 times higher than that for the naturally cooled carrageenan gel. The storage modulus for the rapidly cooled magnetic gel was almost the same as that of the rapidly cooled one. The storage moduli at 0 mT for magnetic gels *G*′ can be written by following Einstein’s equation [23]; *G*′ = *G*′_matrix_(1 + 2.5*ϕ*), where *G*′_matrix_ is the storage modulus of the matrix at 0 mT and *ϕ* is the volume fraction of magnetic particles. Since *ϕ* was constant at 0.12 in this experiment, the storage modulus for the magnetic gel should be 1.3 times higher than that of the matrix irrespective of the cooling rate. The storage moduli for magnetic gels prepared by natural cooling and rapid cooling were 1.6 and 1.2 times higher than those for carrageenan gels, respectively. This suggests that the aggregations of magnetic particles were made in the naturally cooled magnetic gel, and fewer aggregations were made in the rapidly cooled magnetic gel. Figure 2b shows the storage modulus at 50 mT in the linear viscoelastic regime (*γ* = 10^−4^) for magnetic gels prepared by natural cooling and rapid cooling. The storage modulus for the naturally cooled magnetic gel was 5.6 ± 0.4 × 10^4^ Pa, which is 2.7 times higher than that prepared by rapid cooling (=2.1 ± 0.2 × 10^4^ Pa). Figure 2c shows the change in the storage modulus for magnetic gels prepared by natural cooling and rapid cooling. The change in the storage modulus is the difference between the storage moduli at 0 mT *G*′_0_ and at 50 mT *G*′_50_ (Δ*G*′ = *G*′_50_ − *G*′_0_). The change in the storage modulus was 1.9 ± 0.1 × 10^4^ Pa for the naturally cooled magnetic gel and 5.3 ± 1.0 × 10^3^ Pa for the rapidly cooled one. Thus, an interesting result was obtained here, where the cooling rate affected the change in the storage modulus by the magnetic field; the modulus change for the naturally cooled magnetic gel was 3.6 times higher than that of the rapidly cooled one. The change in the storage modulus for the naturally cooled magnetic gel was consistent with the value we previously reported [17].

**Figure 3:**
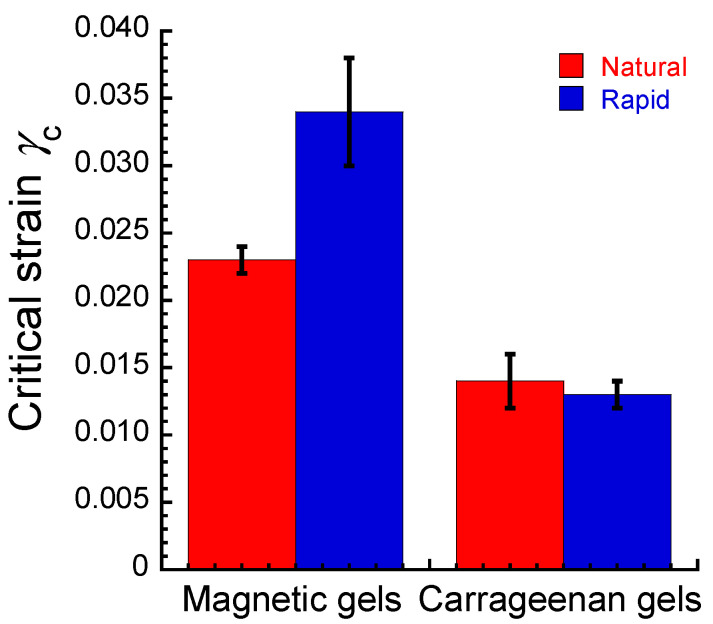
Critical strain for magnetic gels prepared by natural cooling and rapid cooling and their carrageenan gels without magnetic particles under no magnetic field (*f* = 1 Hz).

Figure 3 shows the critical strain at 0 mT *γ*_c_ for magnetic gels and carrageenan gels prepared by natural cooling and rapid cooling. The critical strain is a yield point intersecting the *G*′ and *G*″ in Figure 1, which is the onset of a fluidlike response and has been related to the failure of the network structure [24,25,26]. Accordingly, it can be understood that the critical strain indicates the mechanical strength of the gel network. The critical strains were 0.023 ± 0.001 and 0.034 ± 0.004 for magnetic gels prepared by natural cooling and rapid cooling, respectively. This indicates that the network structure for the rapidly cooled magnetic gel is stronger than that of the naturally cooled one. The critical strain for naturally cooled carrageenan gel was almost the same as that of the rapidly cooled one (~0.012). This strongly indicates that magnetic particles strongly interacted with the carrageenan matrix for the rapidly cooled magnetic gel; it should be noted that rapid cooling does not make the carrageenan network reinforce itself. For the rapidly cooled magnetic gel, it can be considered that the strong interaction between magnetic particles and the carrageenan matrix reduced the change in the storage modulus by the magnetic field.

**Figure 4:**
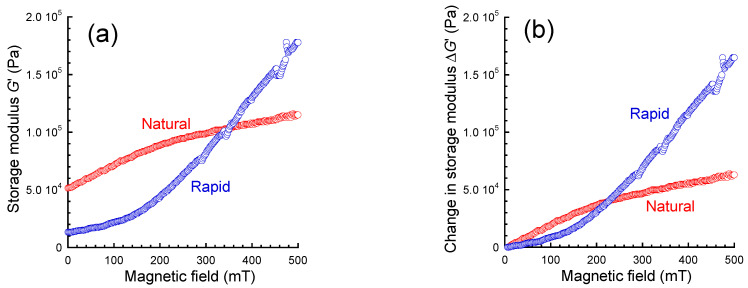
Magnetic field dependence of (**a**) the storage modulus and (**b**) the change in the modulus for carrageenan magnetic gels prepared by natural cooling and rapid cooling (*f* = 1 Hz, *γ* = 10^−4^).

Figure 4a exhibits the magnetic field dependence of the storage modulus at a strain of 10^−4^ for magnetic gels prepared by natural cooling and rapid cooling. The storage modulus for the magnetic gel prepared by natural cooling at 0 mT was 5.2 × 10^4^ Pa and it increased with the magnetic field to 1.1 × 10^5^ Pa at 500 mT. On the other hand, the storage modulus for the rapidly cooled magnetic gel at 0 mT was 1.3 × 10^4^ Pa and it increased with the magnetic field to 1.8 × 10^5^ Pa at 500 mT. An interesting behavior was that the differential coefficient d*G*′/d*B* for the magnetic gel prepared by natural cooling was high at low magnetic fields and it decreased with the magnetic field, which was the opposite of the behavior of typical magnetic soft materials, as seen in the rapidly cooled magnetic gel.

Figure 4b shows the magnetic field dependence of the change in the storage modulus at a strain of 10^−4^ for magnetic gels prepared by natural cooling and rapid cooling. The change in the storage modulus of natural cooling demonstrated a curve with a steep slope at low magnetic fields and a gentle slope at high magnetic fields, as explained in Figure 4a, and reached 6.3 × 10^4^ Pa at 500 mT. The change in the storage modulus for the rapidly cooled magnetic gel demonstrated a curve with a gentle slope at low magnetic fields and a steep slope at high magnetic fields, and reached 1.6 × 10^5^ Pa at 500 mT. In other words, the change in the storage modulus at 500 mT for the rapidly cooled magnetic gel was 2.5 times larger than that of the naturally cooled one. It is very interesting that such a clear difference was observed for these magnetic gels even though the magnetic particle concentration and magnetic field strength were exactly the same. As described in the Introduction, it is usual that the amplitude of the MR effect decreased when increasing the storage modulus at 0 mT, as is the MR response of magnetic elastomers with various plasticizer content [11]. The influence of the cross-linking density on the MR response was reported several years ago [15]; the amplitude of the MR effect decreased with the cross-linking density for polymethyl siloxane gels cross-linked by boric acid. The MR effect of self-healing and printable elastomers, whose elastic modulus changed with temperature, increased with decreasing elastic modulus [27]. Such decreases in the MR response are caused by the reduction in the mobility of magnetic particles within the polymer network. Accordingly, the anomalous response is produced by the special structure of the carrageenan network, which is varied depending on the cooling rate.

**Figure 5:**
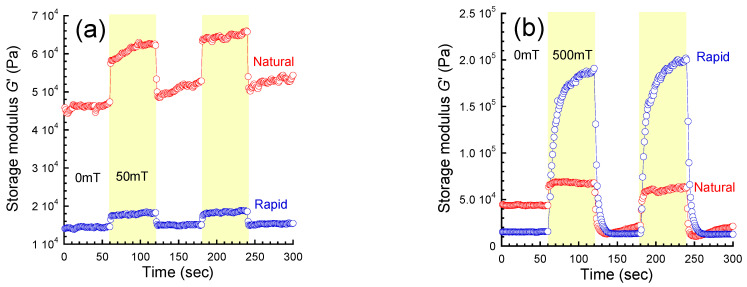
Magnetic field response of storage modulus at (**a**) 50 mT and at (**b**) 500 mT for carrageenan magnetic gels prepared by natural cooling and rapid cooling (*f* = 1 Hz, *γ* = 10^−4^).

Figure 5a demonstrates the magnetic field response of the storage modulus at 50 mT and *γ* = 10^−4^ for magnetic gels prepared by natural cooling and rapid cooling. For both magnetic gels, the storage modulus change was synchronized with the on/off switching of the magnetic field. The storage modulus for the magnetic gel prepared by natural cooling at 0 mT was 4.6 × 10^4^ Pa and it increased to 5.8 × 10^4^ Pa at 50 mT. On the other hand, the storage modulus for the magnetic gel prepared by rapid cooling at 0 mT was 1.4 × 10^4^ Pa and it increased to 1.7 × 10^4^ Pa at 50 mT. By applying the magnetic field repeatedly, the storage modulus at 0 mT for both magnetic gels showed a trend of increasing gradually. This indicates that a certain structure was formed by the application of the magnetic field, and it remained after switching off the field. Figure 5b shows the magnetic field response of the storage modulus at 50 mT and *γ* = 10^−4^ for magnetic gels prepared by natural cooling and rapid cooling. Similar to the response at 50 mT, the storage moduli for both magnetic gels changed in synch with the on/off switching of a magnetic field of 500 mT. The storage modulus at 0 mT for the magnetic gel prepared by natural cooling was 4.5 × 10^4^ Pa and it increased to 6.8 × 10^4^ Pa at 500 mT. On the other hand, the storage modulus at 0 mT for the magnetic gel prepared by rapid cooling was 1.5 × 10^4^ Pa and it gradually increased to 2.0 × 10^5^ Pa at 500 mT. The storage modulus for the magnetic gel prepared by natural cooling apparently decreased with the first application of the magnetic field. This suggests that the original structure was destructed upon the first application of the magnetic field and the broken structure was not further destructed by the subsequent applications of the field. The values of the storage modulus for magnetic gels measured in the switching experiments were consistent with those measured in the strain dependence, as shown in Figure 2.

**Figure 6:**
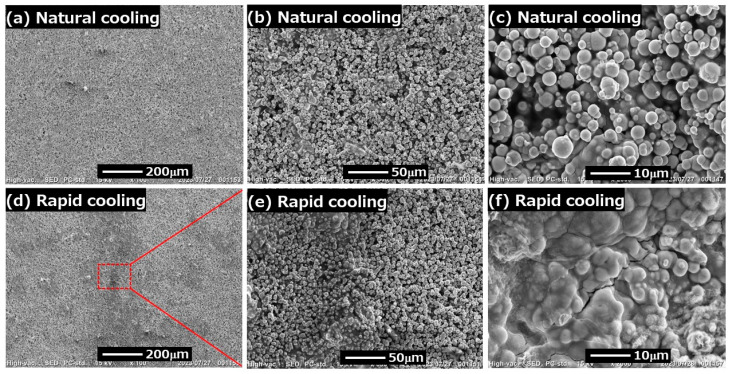
SEM photographs of the surface for dried magnetic gels prepared by natural cooling (**tops**) and rapid cooling (**bottoms**) without magnetic fields.

Figure 6 displays the SEM photographs for magnetic gels prepared by natural cooling and rapid cooling in the absence of magnetic fields. A clear difference in the morphology between magnetic gels prepared by natural cooling and rapid cooling can be seen in photos at low magnification. The morphology for the magnetic gel prepared by natural cooling was heterogeneous, showing many dark parts; meanwhile, that for the magnetic gel prepared by rapid cooling was homogeneous without any dark parts. This feature can clearly be seen in Figure 6b,e. Figure 6e is an enlarged photo of the square indicated by the broken line of Figure 6d. The left half of Figure 6e is the dark part of Figure 6b. That is, the dark part is due not to the heterogeneity of magnetic particles, but to the inhomogeneity of the carrageenan network. Figure 6f clearly shows that the dark part is a continuous phase of the carrageenan in which magnetic particles are embedded, like a chocolate bar with nuts. The continuous phase of the carrageenan was also seen in Figure 6c; however, it was very small, connecting only a few particles. Thus, the area of the continuous phase of carrageenan for the magnetic gel prepared by rapid cooling was far larger than that prepared by natural cooling. It is considered that the movement of magnetic particles in the magnetic gel prepared by rapid cooling is prevented by the continuous phase of the carrageen, which may have resulted in small MR effects, as observed at 50 mT. In contrast, the MR effect at 500 mT for the magnetic gel prepared by rapid cooling was larger than that prepared by natural cooling. It is also considered that the continuous phase of the carrageenan was broken by the movement of magnetic particles on which a strong magnetic force acts.

**Figure 7:**
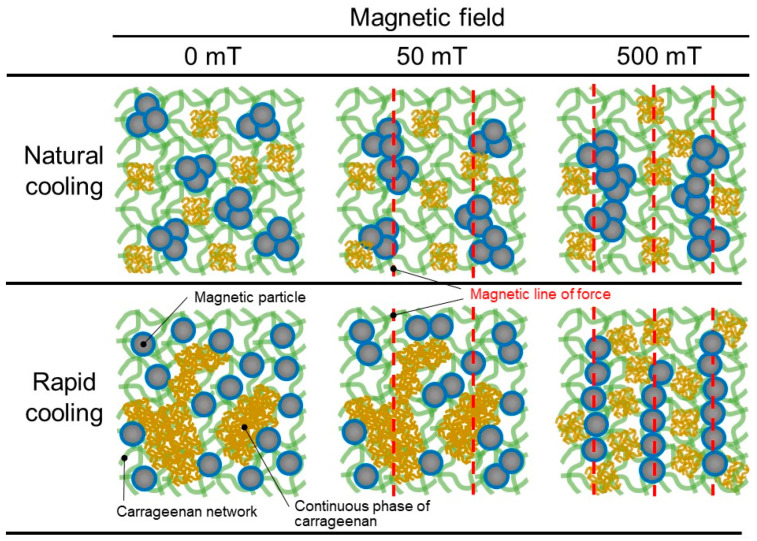
Schematic illustrations representing the morphologies of magnetic particles and carrageenan network at 0, 50, and 500 mT for carrageenan magnetic gels prepared by natural cooling (**tops**) and rapid cooling (**bottoms**).

Figure 7 exhibits the schematic illustrations representing the surface morphologies of magnetic particles and the carrageenan network, and the MR effect for magnetic gels prepared by natural cooling and rapid cooling. The storage moduli at 0 mT for magnetic gels prepared by natural cooling and rapid cooling were higher and lower than those calculated from Einstein’s equation, respectively. Therefore, magnetic particles form aggregations in the magnetic gel prepared by natural cooling and they are dispersed randomly in the magnetic gel prepared by rapid cooling. Large continuous phases of the carrageenan were observed in SEM photographs for the magnetic gel prepared by rapid cooling; some small aggregations of carrageenan were randomly distributed in the magnetic gel prepared by natural cooling. The parameters representing the amplitude of the Payne effect for carrageenan gels prepared by natural cooling and rapid cooling were almost the same, suggesting that the brittleness of the carrageenan network is not affected by the cooling rate. It can be considered under low magnetic fields that magnetic particles in the magnetic gel prepared by rapid cooling are difficult to move due to the obstruction from the continuous phase of the carrageenan. This may result in the MR effect at 50 mT for the magnetic gel prepared by natural cooling being higher than that prepared by rapid cooling. At high magnetic fields (~500 mT), magnetic particles in the magnetic gel prepared by rapid cooling can move and form a chain structure by breaking the restriction from the continuous phase of the carrageenan. The number density of the chains should be high for the magnetic gel prepared by rapid cooling, since magnetic particles are well dispersed as primary particles, which may contribute to a large change in the storage modulus.

## 3. Conclusions

The effect of the cooling rate on the magnetorheological response for carrageenan magnetic hydrogels was investigated using dynamic viscoelastic measurements and morphological observations. At a low magnetic field, the change in the storage modulus for the magnetic gel prepared by natural cooling was higher than that prepared by rapid cooling. Magnetic particles in the magnetic gel prepared by rapid cooling were difficult to move due to the obstruction from the continuous phase of carrageenan. On the other hand, at a high magnetic field, the change in the storage modulus for the magnetic gel prepared by rapid cooling was higher than that prepared by natural cooling. Magnetic particles in the magnetic gel prepared by rapid cooling could move and form a chain structure by breaking the restriction from the continuous phase of the carrageenan. These results coincided with the critical strain, where the magnetic gel prepared by natural cooling has a tough structure compared to that prepared by rapid cooling. The magnetic field dependence of the storage modulus for the magnetic gel prepared by natural cooling showed a curve with a steep slope at low magnetic fields and a gentle slope at high magnetic fields, which was the opposite of that in general magnetic gels. The mechanism should be useful for obtaining materials showing large elasticity changes through weak magnetic fields, and therefore it should be cleared in the future.

## 4. Materials and Methods

### 4.1. Preparation of Magnetic Gels

κ-carrageenan (*M*_w_ = 857 kDa, CS-530, San-Ei Gen F.F.I., Osaka, Japan) was used as a matrix of polysaccharides. The carrageenan was dissolved in pure water at 100 °C for 1 h to prepare the aqueous solution with a concentration of 2.0 wt.%. Carbonyl iron (CS Grade BASF SE., Ludwigshafen am Rhein, Germany) with a diameter of 7.0 µm was used as a magnetic particle. The carbonyl iron particles were dispersed in the carrageenan aqueous solution at 100 °C and then mixed using a mechanical mixer for several minutes to obtain the pre-gel solution. The concentration of magnetic particles was constant at 50 wt.%, which corresponds to a volume fraction of *ϕ* = 0.12. The volume fraction of magnetic particles was determined by the method described in our previous paper [17]. The concentration of magnetic particles in the preparation was the same as that at the final concentration. Immediately after mixing, the pre-gel solution was poured into molds consisting of a silicone spacer and glass plates with a temperature of 50 °C or 4 °C. In this paper, these gels prepared by the hot mold and cold mold are denoted as “magnetic gel of natural cooling” and “magnetic gel of rapid cooling”, respectively. The molds were placed in the refrigerator to allow sufficient time for finishing the gelation. The gelation time for the magnetic gel prepared by natural cooling was within 1 min, and meanwhile the gelation time of that prepared by rapid cooling was within 5 s. It was clearly shown from SEM photographs that no precipitation of magnetic particles occurred for all magnetic gels studied here. The sample was 1 mm in thick and 20 mm in diameter. Carrageenan gels without magnetic particles were also prepared in a similar manner to those with magnetic gels. The diameter of the carbonyl iron was determined to be 7.4 µm ± 0.2 µm using a particle size analyzer (SALD-7000, Shimadzu Co., Ltd., Kyoto, Japan). The saturation magnetization was measured to be 218 emu/g with a SQUID magnetometer (MPMS, Quantum Design Inc., San Diego, CA, USA).

### 4.2. Dynamic Viscoelastic Measurement

The dynamic viscoelastic measurement for the magnetic gels was carried out at a temperature of 20 °C and a frequency of 1 Hz using a rheometer (MCR301, Anton Par Pty Ltd., Graz, Austria) with an electromagnetic system (PS-MRD) and a non-magnetic parallel plate (PP20/MRD). The strain varied from 10^−5^ to 1. The normal force initially applied was approximately 0.3 N. For the strain-sweep measurement shown in Figure 1, a constant magnetic field of 50 mT was applied. The magnetic field was swept from 0 to 500 mT for the measurement of Figure 4. A pulsatile magnetic field of 0 and 50 mT was applied for the experiment in Figure 5. In a magnetic field of 50 mT, the magnetic force acting on magnetic particles is weak and the movement of magnetic particles is affected by the viscoelasticity of the matrix. In other words, the information relating to the viscoelasticity of the matrix can be obtained from the amplitude of elasticity changes by the magnetic field [17]. On the other hand, in a magnetic field of 500 mT, the magnetic force acting on magnetic particles is strong and the movement of magnetic particles is not influenced by the viscoelasticity of the matrix [16]. The magnetic field strength at the sample stage in the rheometer was measured using a Gauss meter (TM-601, Kanetec Co., Ltd., Nagano, Japan). The magnetic field strength changed from 0 to 500 mT when the excitation current was varied from 0 to 3.0 A. The mean values and standard errors of the storage and loss moduli for three different samples from one batch were evaluated.

### 4.3. Scanning Electron Microscope Observations

To observe the dispersion of magnetic particles and the morphology of the carrageenan network, the surface of dried magnetic gels was observed using a scanning electron microscope (SEM) (JCM-6000 Neoscope, JEOL Ltd. Tokyo, Japan) at an acceleration voltage of 15 kV.

## Data Availability

Not applicable.
